# Host cell species-specific effect of cyclosporine A on simian immunodeficiency virus replication

**DOI:** 10.1186/1742-4690-9-3

**Published:** 2012-01-06

**Authors:** Hiroaki Takeuchi, Hiroshi Ishii, Tetsuya Kuwano, Natsuko Inagaki, Hirofumi Akari, Tetsuro Matano

**Affiliations:** 1 Department of Molecular Virology, Tokyo Medical and Dental University, Tokyo, Japan; 2 The Institute of Medical Science, The University of Tokyo, Tokyo, Japan; 3 AIDS Research Center, National Institute of Infectious Diseases, Tokyo, Japan; 4 Center for Human Evolution Modeling Research, Primate Research Institute, The University of Kyoto, Aichi, Japan

**Keywords:** HIV-1, SIV, cyclophilin A, cyclophilin B, cyclosporine A, tropism

## Abstract

**Background:**

An understanding of host cell factors that affect viral replication contributes to elucidation of the mechanism for determination of viral tropism. Cyclophilin A (CypA), a peptidyl-prolyl *cis-trans *isomerase (PPIase), is a host factor essential for efficient replication of human immunodeficiency virus type 1 (HIV-1) in human cells. However, the role of cyclophilins in simian immunodeficiency virus (SIV) replication has not been determined. In the present study, we examined the effect of cyclosporine A (CsA), a PPIase inhibitor, on SIV replication.

**Results:**

SIV replication in human CEM-SS T cells was not inhibited but rather enhanced by treatment with CsA, which inhibited HIV-1 replication. CsA treatment of target human cells enhanced an early step of SIV replication. CypA overexpression enhanced the early phase of HIV-1 but not SIV replication, while CypA knock-down resulted in suppression of HIV-1 but not SIV replication in CEM-SS cells, partially explaining different sensitivities of HIV-1 and SIV replication to CsA treatment. In contrast, CsA treatment inhibited SIV replication in macaque T cells; CsA treatment of either virus producer or target cells resulted in suppression of SIV replication. SIV infection was enhanced by CypA overexpression in macaque target cells.

**Conclusions:**

CsA treatment enhanced SIV replication in human T cells but abrogated SIV replication in macaque T cells, implying a host cell species-specific effect of CsA on SIV replication. Further analyses indicated a positive effect of CypA on SIV infection into macaque but not into human T cells. These results suggest possible contribution of CypA to the determination of SIV tropism.

## Background

Viral replication is modulated by host cell factors, with the species specificity of these factors affecting viral tropism. Some of these host factors can restrict viral replication. The anti-viral systems mediated by such host restriction factors, termed intrinsic immunity, play an important role in determining species-specific barriers against viral infection. For instance, Fv-1 in mice is known to restrict replication of a murine leukemia virus [1-3]; and tripartite interaction motif 5α (TRIM5α) recently has been found to be responsible for restricting human immunodeficiency virus type 1 (HIV-1), but not simian immunodeficiency virus (SIV) infection in old world monkey (OWM) cells [4-9]. Restriction of retroviral replication by these host cell factors takes place after viral entry, but before the integration step; and the viral determinants for this type of restriction have been mapped to the capsid (CA) protein [1,3,10-12]. Understanding how host cell factors affect viral replication, positively or negatively, would contribute to elucidating the molecular mechanism that determines viral tropism.

Cyclophilin A (CypA), a peptidyl-prolyl isomerase, is a host cell factor essential for efficient HIV-1 replication in human cells [13-19]. CypA promotes an early step in HIV-1 replication, after viral entry but before reverse transcription [20]. Late in replication, during virus assembly, CypA is incorporated into progeny HIV-1 virions through CypA interaction with viral CA [13,14,18]. Disruption of CypA incorporation by Gag mutations or by treatment of infected cells with cyclosporine A (CsA), a PPIase inhibitor, results in a reduction in the infectivity of the progeny viruses produced [13,14,16,21-24]. Furthermore, interaction of viral CA with CypA in target cells after viral entry has been shown to promote HIV-1 replication, indicating the importance of target cell CypA for an early step of HIV-1 replication in human cells [15,19,25,26].

In contrast, the effect of CypA on SIV replication has not been well-documented, although a possible interaction between CypA and SIV CA has been indicated [17,27,28]. A recent study has shown a suppressive effect of CypA on replication of *vif*-deleted SIV in human Jurkat cells, which was counteracted by SIV Vif inhibiting CypA incorporation during virus assembly [27]. This Vif function can be distinguished from its anti-human APOBEC3G (apolipoprotein B mRNA-editing enzyme-catalytic subunit 3G) function.

In the present study, we have investigated the effect of CsA on wild-type SIV replication in human or macaque T cells. SIV replication in human T cells was not inhibited but rather enhanced by treatment with CsA, which inhibits HIV-1 replication. In contrast, CsA treatment abrogated SIV replication in macaque T cells, indicating a species-specific effect of CsA on SIV replication. CypA knock-down or overexpression suggested that CypA affects SIV replication differently in human and macaque T cells and suggested possible contribution of CypA to the determination of SIV tropism.

## Results

### Effect of CsA treatment on SIV replication in human T cells

**Figure 1:**
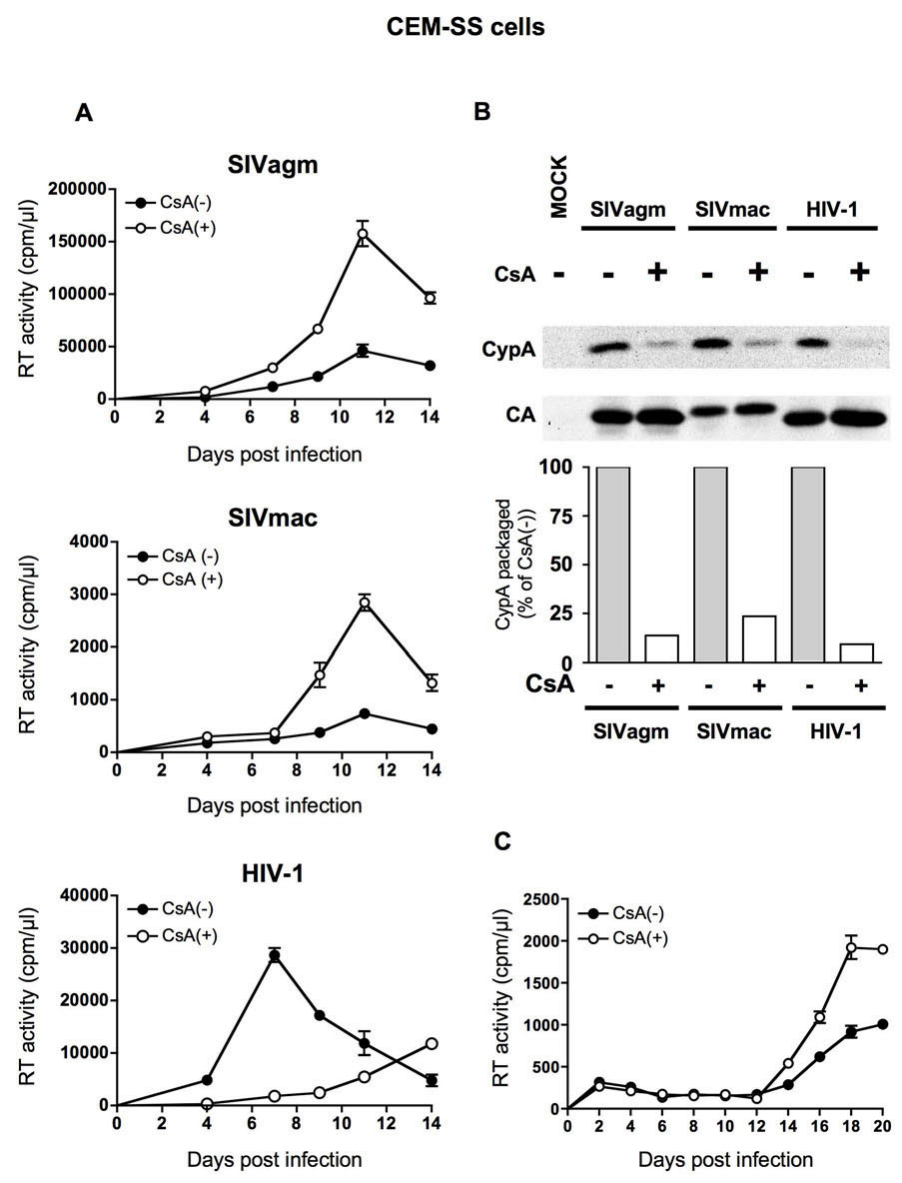
**SIV and HIV-1 replication in human cells**. (A) SIV and HIV-1 replication kinetics in CEM-SS cells. CEM-SS cells were infected with SIVagm (top panel), SIVmac (middle panel), and HIV-1 (bottom) in the absence (CsA[-], closed circles) or presence of 2.5 μM CsA (CsA[+], open circles). Virus production was monitored by measuring RT activity in the culture supernatants. (B) CypA incorporation into virions. Virus-containing supernatants were harvested from CsA-untreated and CsA-treated CEM-SS cells infected with SIVagm, SIVmac and HIV-1. A mock-infected sample was included as a control. CypA-specific band densities were quantified by densitometric scanning and are plotted in the lower panel. For each virus, the density of the band from CsA-untreated cells was defined as 100% and the ratio (%) of the density of the band from CsA-treated cells to that from CsA-untreated cells was calculated. The image of one representative blot is shown. (C) SIVagm replication kinetics in A3.01 cells.

We investigated the effect of CsA treatment on wild-type SIV replication in human CEM-SS T cells. Replication of wild-type SIVagm, SIVmac, and HIV-1 in the presence of CsA was compared with that in the absence of CsA (Figure 1A). Consistent with previous reports, CsA treatment inhibited the packaging of CypA into HIV-1 particles (Figure 1B) and impaired HIV-1 replication in CEM-SS cells (Figure 1A). CypA was incorporated into SIVagm and SIVmac progeny virions, although not efficiently, and CsA treatment further abrogated this low level of CypA incorporation (Figure 1B) without the reduction of endogenous CypA (data not shown). Interestingly, however, CsA treatment did not inhibit but rather enhanced SIV replication in CEM-SS cells (Figure 1A). This CsA-mediated enhancement of SIV replication was also observed in human A3.01 T cells (Figure 1C).

### Effect of CsA treatment of target human T cells on SIV infection

**Figure 2:**
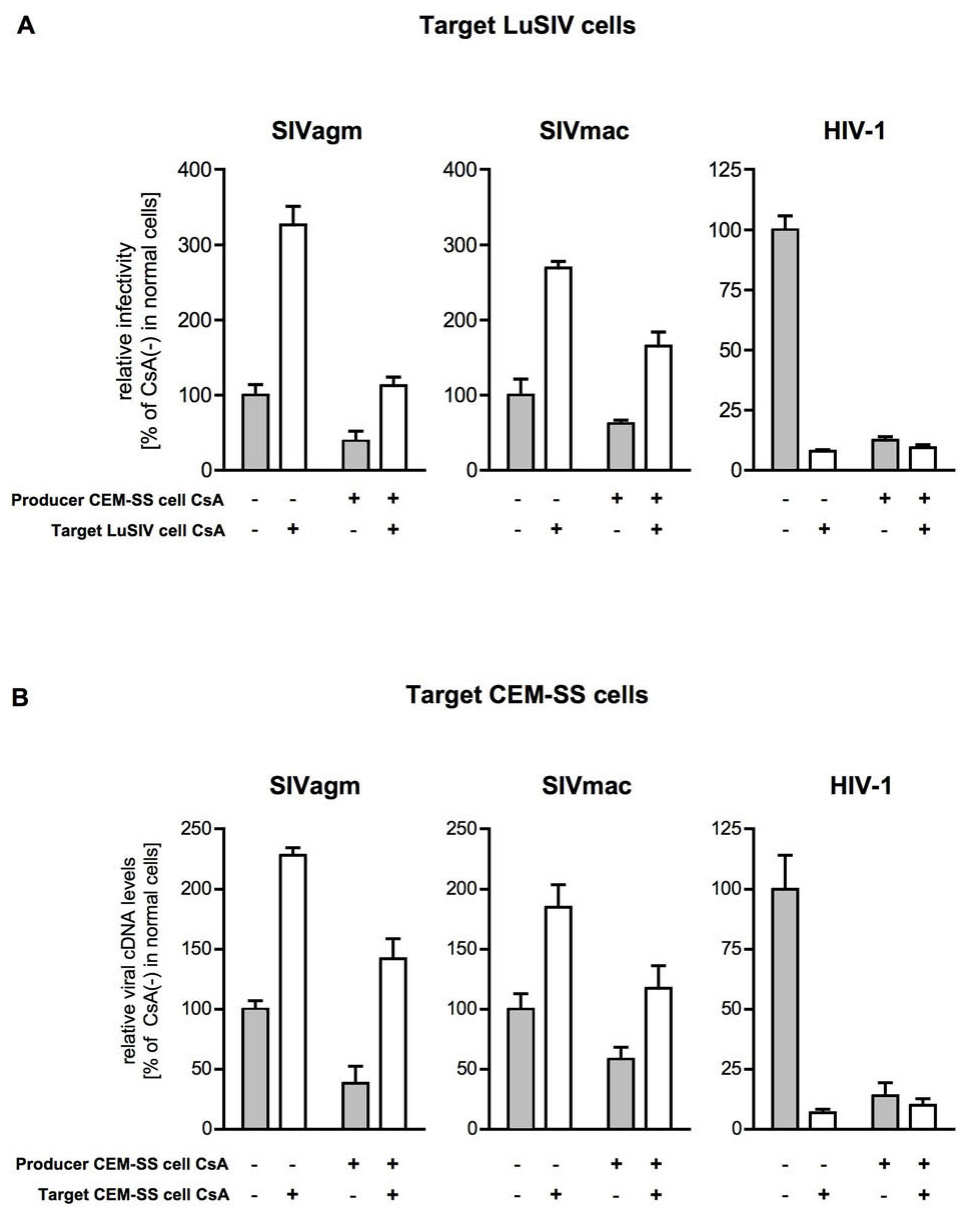
**Effect of CsA treatment of target human T cells on SIV infection**. (A) Effect of CsA treatment of producer human CEM-SS or target human LuSIV cells on viral infectivity. SIVagm, SIVmac, and HIV-1 from producer CEM-SS cells in the absence (Producer CEM-SS cell CsA [-]) or presence of CsA (Producer CEM-SS cell CsA [+]) were used to infect CsA-untreated (Target LuSIV cell CsA [-]) or CsA-treated target LuSIV cells (Target LuSIV cell CsA [+]). Luciferase activity in target LuSIV cells was measured 24 hr after infection. Relative infectivity is shown as the ratio (%) of the luciferase activity to that of viruses produced from CsA-untreated CEM-SS in CsA-untreated LuSIV cells. Mean values and standard deviations in four independent experiments are shown. (B) Effect of CsA treatment of producer or target human cells on viral replication. SIVagm, SIVmac, and HIV-1 from producer CEM-SS cells in the absence (Producer cell CsA [-]) or presence of CsA (Producer cell CsA [+]) were used to infect CsA-untreated (Target cell CsA [-]) or CsA-treated target CEM-SS cells (Target cell CsA [+]). Heat-inactivated virus was used as an infection control. Relative viral cDNA levels are shown as the ratio (%) of the viral cDNA levels to that of virus produced from CsA-untreated CEM-SS in CsA-untreated CEM-SS cells. Mean values and standard deviations in six independent experiments are shown.

Recent studies have indicated that CypA in target cells is crucial for an early HIV-1 replication step in human cells [15,19,25,26,29]. We therefore studied the effect of CsA treatment of target cells on SIV infection. Viruses were produced from CsA-untreated or CsA-treated CEM-SS cells and used to infect CsA-untreated or CsA-treated target human LuSIV cells. Cell lysates were prepared from the target LuSIV cells 24 h post-infection, and luciferase activity was measured to assess the efficiency of SIV infection (Figure 2A). Similar to the results in Jurkat cells [27], CsA treatment of either the producer cells or the target cells resulted in suppression of HIV-1 infection confirming the importance of CypA both in producer and target human cells for efficient HIV-1 replication [16,30]. In contrast, SIVagm infection was not inhibited but rather enhanced by CsA treatment of target cells although it was decreased by CsA treatment of producer cells. Similarly, SIVmac infection was not inhibited by CsA treatment of target cells but decreased by CsA treatment of producer cells.

Infection efficiency was also determined by measuring the amounts of viral cDNA synthesized in target CEM-SS cells after viral infection by quantitative PCR (Figure 2B). As a negative control, cells were also infected with heat-inactivated viruses. Consistent with the above results (Figure 2A), the amounts of viral cDNA synthesized after HIV-1 infection were reduced by CsA treatment of target cells (Figure 2B). In contrast, CsA-mediated effect on viral cDNA synthesis after SIV infection into CEM-SS cells (Figure 2B) was consistent with the results shown in Figure 2A. CsA treatment did not inhibit SIV infection in another human T cells, A3.01, either (data not shown). Thus, CsA treatment of human producer cells reduced infectivity of progeny SIVs, whereas CsA treatment of human target cells did not inhibit but rather enhanced SIV infection.

### Effect of CypA knock-down on SIV replication in human T cells

**Figure 3:**
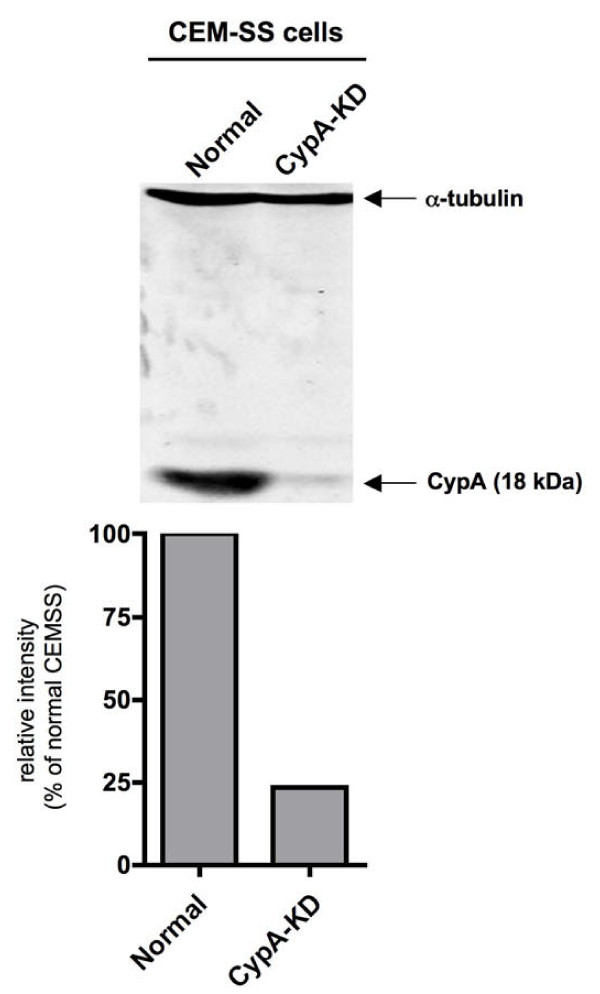
**Immunoblot analysis of CypA expression**. Lysates of CEM-SS (normal), CypA-KD cells were subjected to the analysis using anti-CypA antibodies. Anti-α-tubulin antibody was used as loading control. The ratio (%) of CypA band density in CypA-KD to that in normal CEM-SS is shown at the bottom panels. The image of one representative blot is shown.

**Figure 4:**
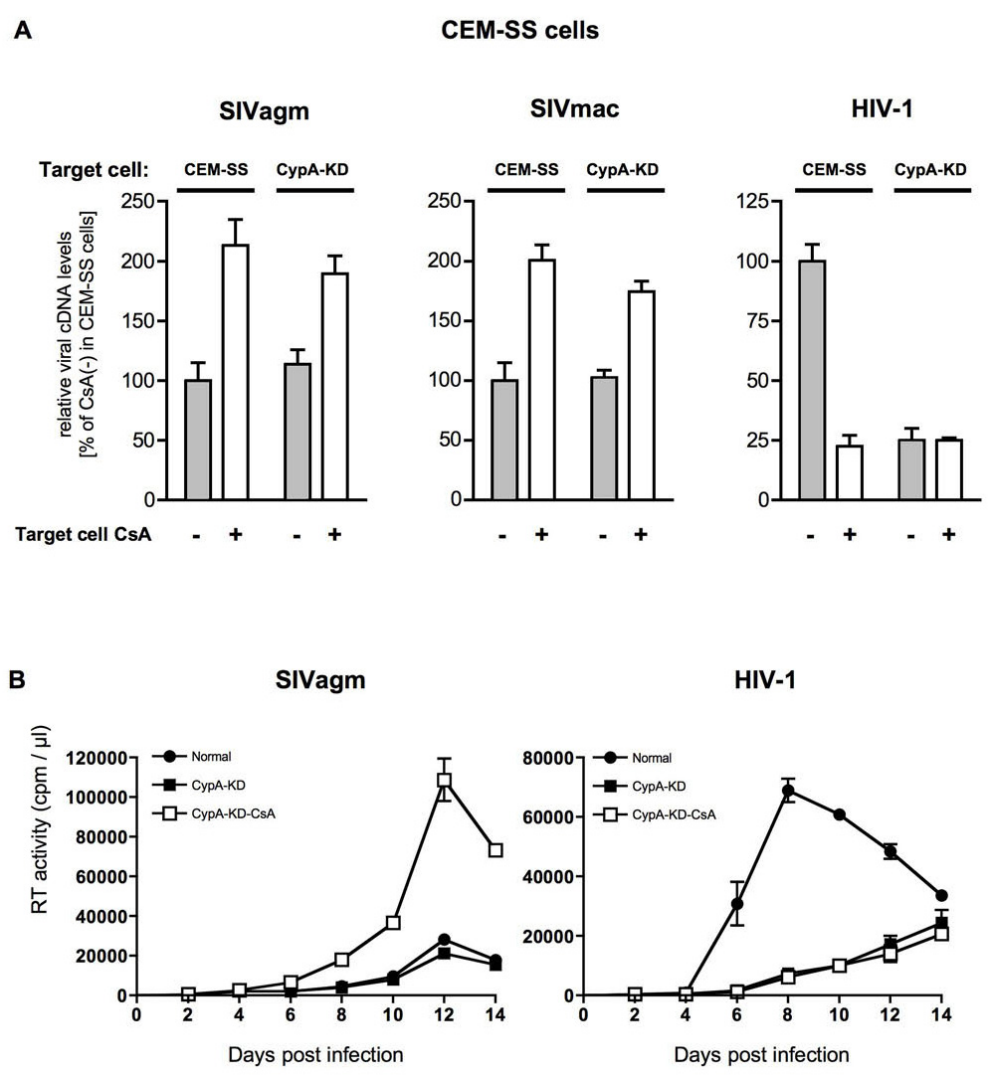
**Effect of CypA knock-down on SIV and HIV-1 replication in human CEM-SS cells**. (A) The amounts of viral cDNA synthesized after SIVagm (left panel), SIVmac239 (middle panel) or HIV-1 (right panel) infection. Viruses produced from CEM-SS cells were used to infect CsA-untreated or CsA-treated target CEM-SS, CypA-KD cells. Heat-inactivated virus was used as an infection control. The synthesized viral cDNA levels were measured by real-time PCR. Mean values and standard deviations in six independent experiments are shown. Relative viral cDNA levels are shown as the ratio (%) of the viral cDNA levels to that of viruses produced from CsA-untreated CEM-SS in CsA-untreated CEM-SS cells. (B) Replication of SIVagm (left panels) or HIV-1 (right panels) in CypA-KD CEM-SS cells. Viral production in normal CsA-untreated CEM-SS (closed circles), CsA-untreated (closed squares) or CsA-treated CypA-KD (open squares) was monitored by measuring RT activity in the culture supernatants.

To examine the effect of CypA on SIV replication in CEM-SS cells, we established CypA knocked-down (CypA-KD) CEM-SS cell lines. CypA expression in CEM-SS CypA-KD cells was stably suppressed by CypA-specific shRNA (Figure 3). We confirmed that both cell proliferation and cell surface levels of CD4 and CXCR4 that are required for HIV-1 entry showed no difference between parental CEM-SS and CypA-KD CEM-SS cells (data not shown). As previously reported [16], CypA knock-down reduced viral cDNA synthesis after HIV-1 infection (Figure 4A) and inhibited HIV-1 replication (Figure 4B). In CypA-KD CEM-SS cells, CsA treatment showed little effect on viral cDNA synthesis after HIV-1 infection, indicating that CypA inhibition was largely involved in CsA-mediated reduction of HIV-1 infection. In contrast, CypA knock-down did not reduce viral cDNA synthesis after SIV infection (Figure 4A) or SIV replication (Figure 4B). These results indicate that target cell CypA is essential for HIV-1 infection but not required for SIV infection into human T cells. CsA treatment, however, enhanced SIV replication even in CypA-KD cells (Figure 4A and 4B, SIVagm), suggesting the possibility that CsA neutralizes the residual CypA population which remains in the CypA-KD cells as shown by Western blotting in Figure 3, or the possible involvement of another host factor in this CsA-mediated enhancement of SIV replication. Similar to the results obtained by replication in CypA-KD CEM-SS cells, the effect of CypA knock-down on SIV infection or SIV replication was also observed in A3.01 cells (data not shown).

**Figure 5:**
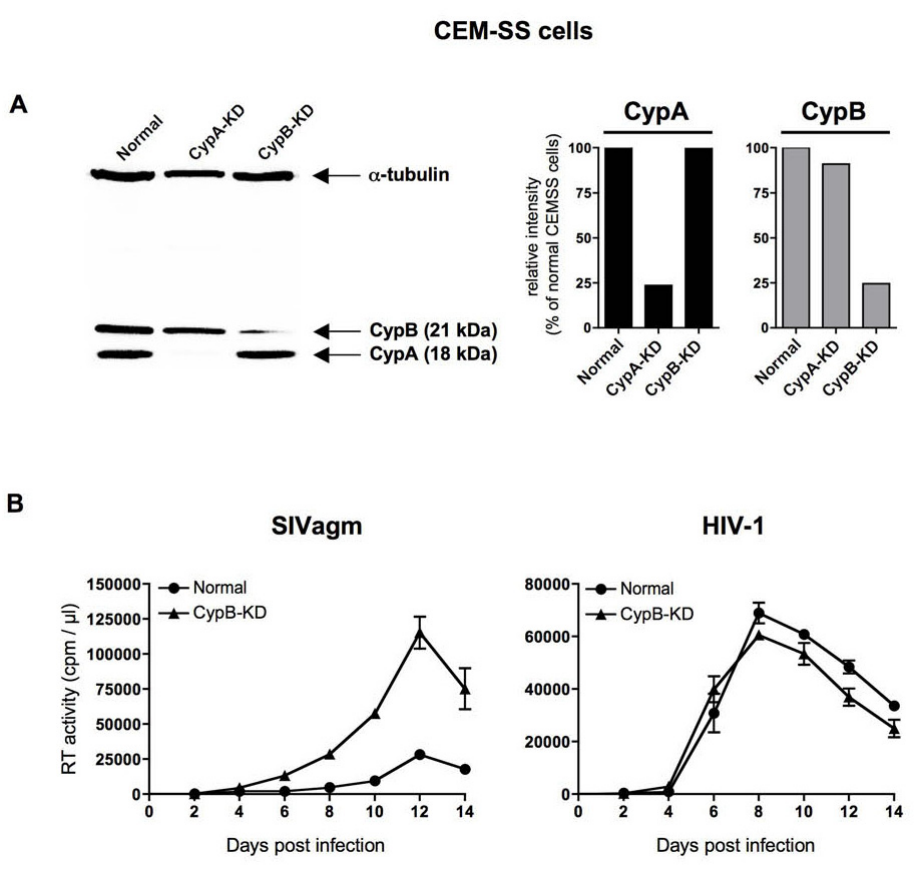
**Effect of CypB knock-down on HIV-1 and SIV replication in human CEM-SS T cells**. (A) Immunoblot analysis of CypB expression. Lysates of CEM-SS (normal), CypA-KD, and CypB-KD cells were subjected to the immunoblot analysis using anti-α-tubulin, anti-CypA and anti-CypB antibodies (Abcam Inc., Cambridge, MA) (left panel). CypA- and CypB-specific band densities were quantified by densitometric scanning and are plotted in the right panels. The image of one representative blot is shown. (B) Replication of SIVagm (left panels) or HIV-1 (right panels) in CypB-KD CEM-SS cells. Viral production in normal CEM-SS (closed circles) or CypB-KD cells (closed triangles) was monitored by measuring RT activity in the culture supernatants.

We also established a cyclophilin B (CypB), another PPIase, knocked-down (CypB-KD) CEM-SS cell line (Figure 5A). Overall SIV replication was enhanced in CypB-KD cells (Figure 5B, SIVagm). As predicted, HIV-1 replication was not affected by the CypB knock-down (Figure 5B, HIV-1). These results suggest that the CsA-induced enhancement of SIV replication in human T cells observed in Figure 1A is mediated largely if not exclusively by an inhibition of CypB.

### Effect of CsA treatment on SIV replication in macaque T cells

**Figure 6:**
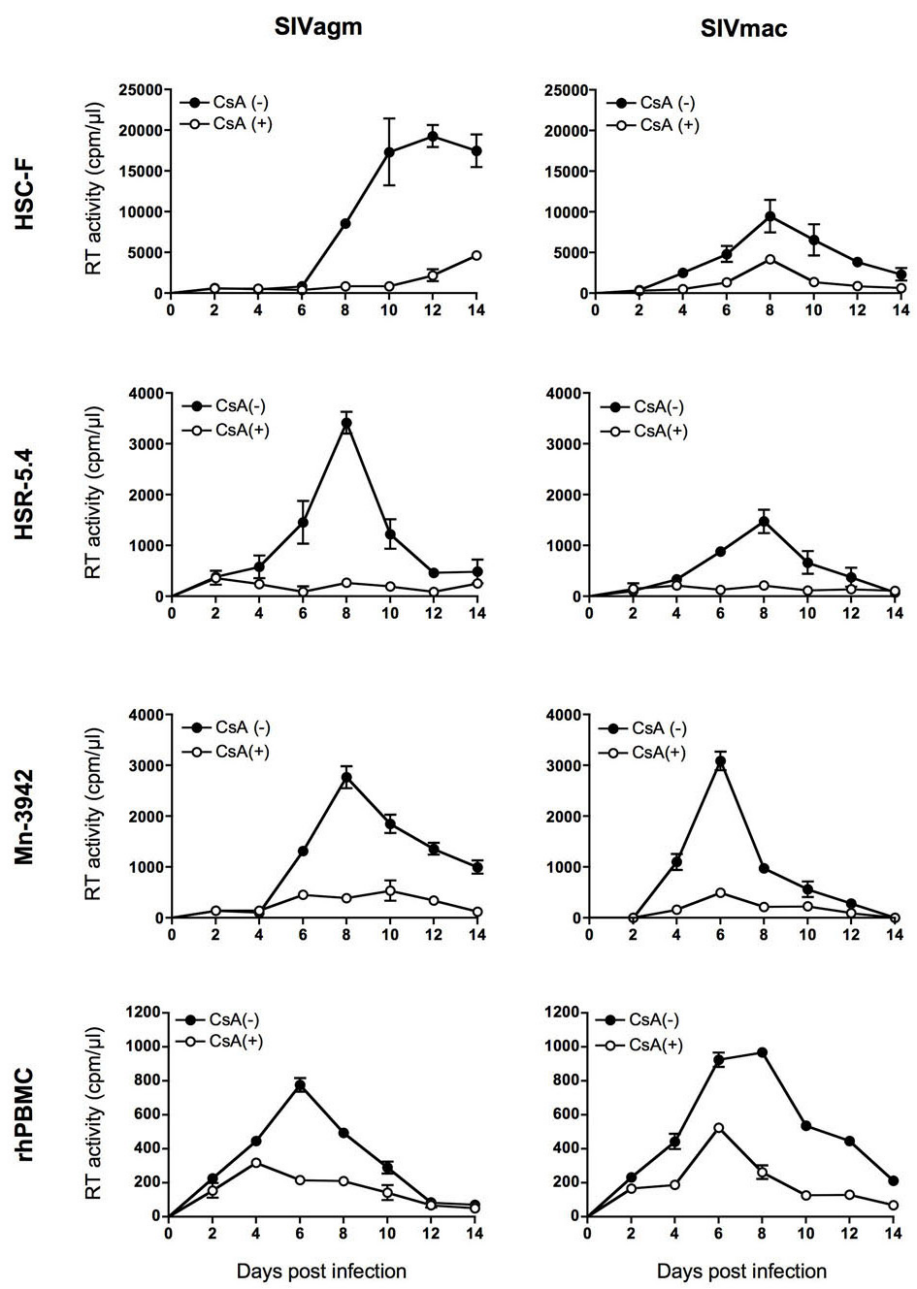
**SIV replication in macaque cells**. Cynomolgus macaque HSC-F, rhesus macaque HSR-5.4, pig-tailed macaque Mn-3942, and rhesus macaque PBMCs (rhPBMC) were infected with SIVagm (left panels) and SIVmac (right panels) and cultured in the absence (CsA[-], closed circles) or presence of 2.5 μM CsA (CsA[+], open circles). Virus production was monitored by measuring RT activity in the culture supernatants.

**Figure 7:**
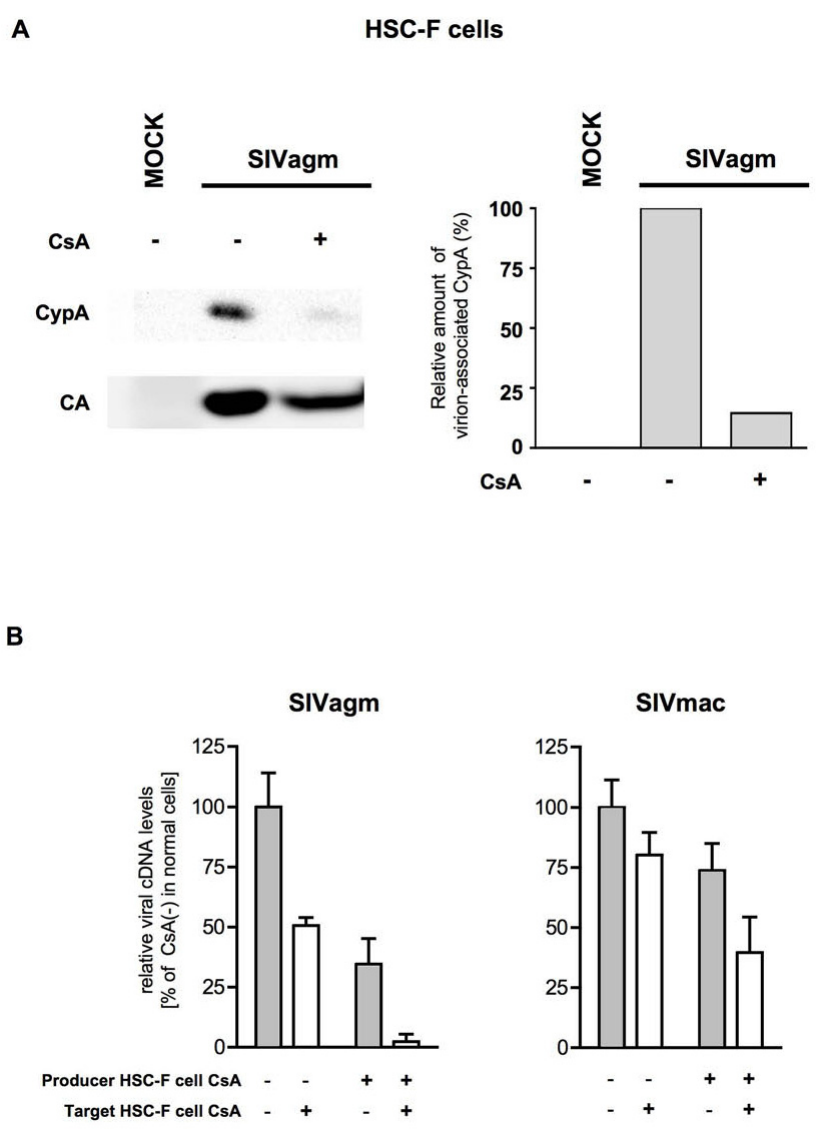
**Effect of CsA treatment of macaque T cells on SIV infection**. (A) Efficiency of CypA incorporation into virions from producer macaque HSC-F cells. Culture supernatants were harvested from CsA-untreated mock, and CsA-untreated and CsA-treated HSC-F cells infected with SIVagm. The CypA incorporation efficiency (right panel) is shown as described in the legend for Figure 1B. The image of one representative blot is shown. (B) Effect of CsA treatment of producer or target macaque HSC-F cells on SIV replication. SIVagm and SIVmac produced from HSC-F cells in the absence (Producer cell CsA [-]) or presence of CsA (Producer cell CsA [+]) was used to infect CsA-untreated (Target cell CsA [-]) or CsA-treated target HSC-F cells (Target cell CsA [+]). Heat-inactivated virus was used as an infection control. Relative viral cDNA levels are shown as the ratio (%) of the viral cDNA levels to that of virus produced from CsA-untreated HSC-F in CsA-untreated HSC-F cells. The synthesized viral cDNA levels were measured by real-time PCR. Mean values and standard deviations in four independent experiments are shown.

We next examined the effect of CsA treatment on SIV replication in macaque cells using three macaque T-cell lines: cynomolgus macaque-derived HSC-F, rhesus macaque-derived HSR-5.4, and pig-tailed macaque-derived Mn-3942 (Figure 6). Both SIVmac239 and SIVagm replicated well in all three cell lines, with the most efficient replication in HSC-F cells. However, in contrast to the results above for SIV replication in human T cells, CsA treatment inhibited SIVagm replication in all three macaque T cell lines. This inhibitory effect of CsA on SIV replication was also observed in rhesus macaque PBMCs (Figure 6). The CsA effect on HSC-F cell proliferation was marginal and not considered as responsible for this inhibition of SIV replication. Indeed, treatment of these macaque cells with lower concentration (0.5 μM) of CsA had no effect on cell proliferation but resulted in inhibition of SIV replication (data not shown). CypA was incorporated into SIV virions in the absence of CsA, but its incorporation was inhibited by CsA treatment of HSC-F cells (Figure 7A) without the reduction of endogenous CypA (data not shown). HIV-1 replication was undetectable in these macaque T-cell lines even in the presence of CsA (data not shown), although the possibility of enhancement of HIV-1 infection by CsA treatment in OWM cell lines has been indicated previously [28,31,32]. Thus, SIV replication in macaque T cells is inhibited by CsA treatment, indicating the exact opposite effect of CsA on SIV replication in macaque T cells than in human T cells.

We then examined the effect of CsA treatment of producer or target macaque HSC-F cells on SIV infection (Figure 7B). Measurement of synthesized viral cDNA levels in target cells infected with SIV from CsA-untreated or CsA-treated producer HSC-F cells showed that SIV from CsA-treated producer HSC-F cells had lower amount than that from CsA-untreated producer HSC-F cells. In contrast to the results in human CEM-SS cells (Figure 2B), CsA treatment of target HSC-F cells did not increase viral cDNA synthesis after SIV infection, but rather resulted in a reduction in synthesis. Thus, CsA treatment of macaque T cells has an inhibitory effect on a post-entry step of SIV replication.

### Effect of exogenous CypA on SIV infection in human and macaque T cells

Our attempts to knock down CypA in macaque HSC-F cells were unsuccessful. We therefore examined and compared the effect of CypA overexpression in target CEM-SS and HSC-F cells on SIV infection. We first examined the effect of exogenously expressed CypA in human target cells on SIV replication. CEM-SS or CypA-KD cells were transfected (nucleofected) with plasmids expressing HA-control or CypA-HA, respectively. Transfected cells were enriched by magnetic beads sorting as described in Methods. More than 97% of sorted cells were shown to express the marker protein (H-2K^k^) without a reduction in cell-surface CD4 levels (data not shown).

**Figure 8:**
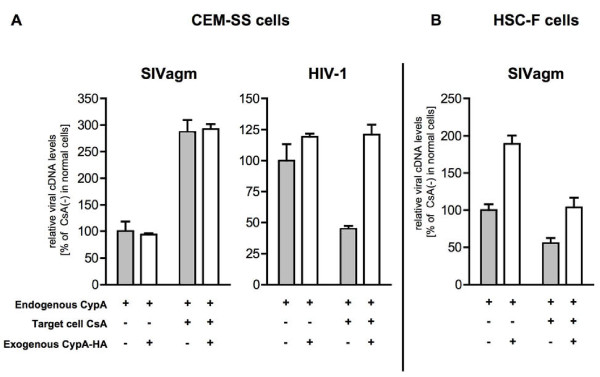
**Effect of exogenous CypA expression on SIV and HIV-1 replication**. (A) The amounts of viral cDNA synthesized after SIVagm (left panel) or HIV-1 (right panel) infection in human CEM-SS cells. Viruses produced from normal CEM-SS cells were used to infect CsA-untreated or CsA-treated target normal CEM-SS cells. Heat-inactivated virus was used as an infection control. Relative viral cDNA levels are shown as the ratio (%) of the viral cDNA levels to that of viruses produced from CsA-untreated CEM-SS in CsA-untreated CEM-SS cells. Mean values and standard deviations in three independent experiments are shown. (B) The amounts of viral cDNA after SIVagm infection in macaque HSC-F cells. SIVagm produced from normal HSC-F cells was used to infect CsA-untreated or CsA-treated target normal HSC-F cells. Heat-inactivated virus was used as an infection control. Relative viral cDNA levels are shown as the ratio (%) of the viral cDNA levels to that of viruses produced from CsA-untreated HSC-F in CsA-untreated HSC-F cells. Mean values and standard deviations in three independent experiments are shown.

We found that exogenous CypA increased HIV-1 cDNA synthesis in CsA-treated CEM-SS cells (Figure 8A), confirming the positive effect of target cell CypA on the early phase of HIV-1 replication. However, SIV infection was not affected by exogenous CypA expression even in CsA-treated CEM-SS cells (Figure 8A). In contrast, CypA overexpression in HSC-F target cells did not reduce but rather enhanced viral cDNA synthesis after SIV infection (Figure 8B). These results suggest that target cell CypA essential for HIV-1 infection is not largely involved in SIV infection in human T cells but has a positive effect on SIV replication in macaque T cells.

### Effect of CsA treatment of target cells on human cell- or macaque cell-derived SIV infection

**Figure 9:**
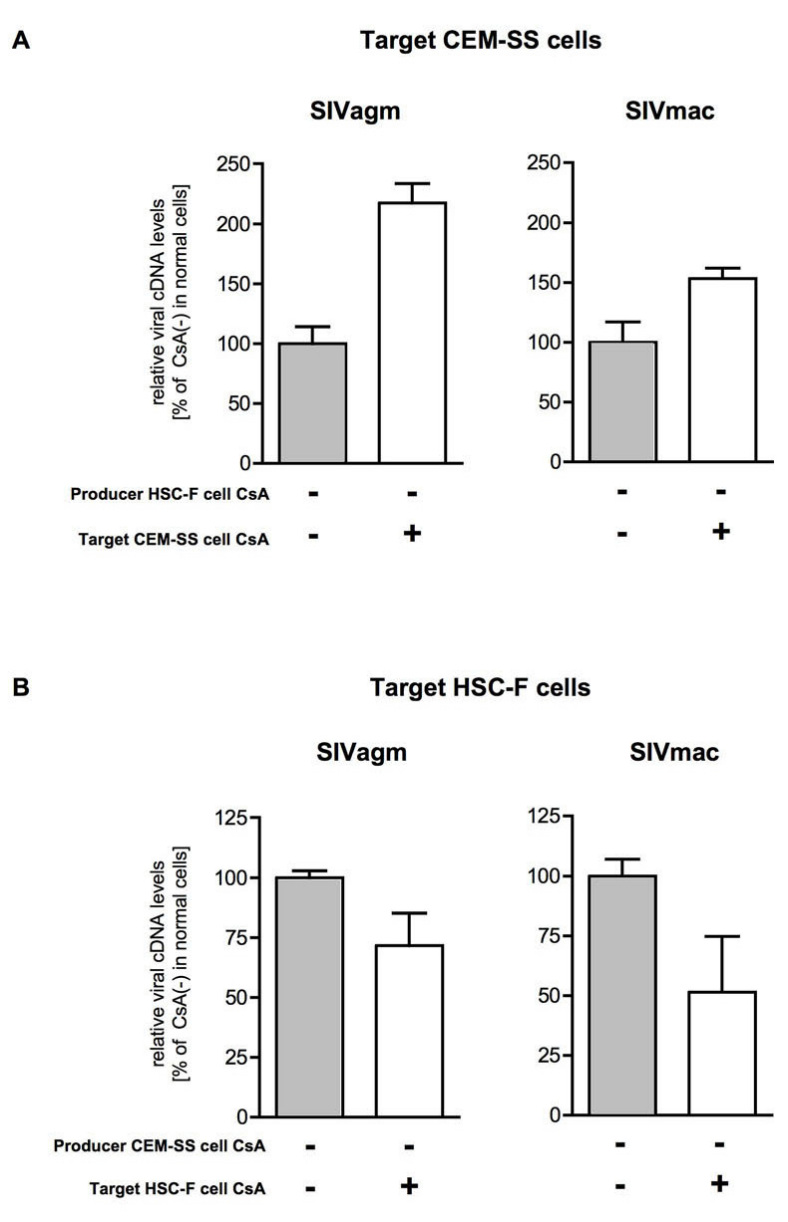
**Infection of human and macaque cells with macaque- and human-derived SIV**. (A) SIVagm and SIVmac produced from HSC-F cells in the absence or presence of CsA were used to infect CsA-untreated or CsA-treated target CEM-SS cells. Relative viral cDNA levels are shown as the ratio (%) of the viral cDNA levels to that of viruses produced from CsA-untreated HSC-F in CsA-untreated CEM-SS cells. Mean values and standard deviations in three independent experiments are shown. (B) SIVagm and SIVmac produced from CEM-SS cells in the absence or presence of CsA were used to infect CsA-untreated or CsA-treated target HSC-F cells. Relative viral cDNA levels are shown as the ratio (%) of the viral cDNA levels to that of viruses produced from CsA-untreated CEM-SS in CsA-untreated HSC-F cells. The relative viral cDNA levels synthesized were measured by real-time PCR. Mean values and standard deviations in three independent experiments are shown.

We then investigated how the infectivity of SIV from producer macaque cells is affected by CsA treatment of target human cells and how the infectivity of SIV from producer human cells is affected by CsA treatment of target macaque cells. We first measured viral cDNA levels in target human CEM-SS cells after infection with SIV from producer macaque HSC-F cells (Figure 9A). Similar to the results obtained by infection of CEM-SS cells with CEM-SS-derived SIV (Figure 2), CsA treatment of target CEM-SS cells enhanced infection by HSC-F-derived SIV. These data indicate that even macaque cell-derived SIV infection is enhanced by CsA treatment of target human cells. Finally, we examined the effect of CsA on the infectivity of CEM-SS-derived SIV in macaque HSC-F cells (Figure 9B). The results were similar to those for infection of HSC-F cells by HSC-F-derived SIV (Figure 7B), i.e. CsA treatment of either producer or target cells diminished infectivity of CEM-SS-derived SIV in HSC-F cells, although previous reports showed that the role of virion-associated CypA is minor compared to that of target cell CypA [15,19,25,26,29].

## Discussion

The present study showed that CsA treatment, which inhibits HIV-1 replication, does not inhibit but rather enhances SIV (SIVmac239 and SIVagm) replication in human T cells. In SIV infection of both CEM-SS and A3.01 cells, CsA treatment resulted in production of viruses with lower infectivity but enhanced an early step of replication. The present study indicates that CsA treatment exerts different effects on the early phase of HIV-1 and SIV replication in human T cells. Indeed, previous study also showed the positive effect of CsA on HIV-1 and SIV vectors infection in primary mouse cells, suggesting different effects of CsA on the early phase of HIV-1 between human and mouse cells [33]. Additionally, we found that expression level of CCR5 on cell surface was lower than that of CXCR4 in CEM-SS cells (data not shown). This may be due to different kinetics between CCR5-tropic SIV and CXCR4-tropic HIV-1 in Figure 1A.

Knock down of CypA, an essential host factor for HIV-1 replication, did not reduce SIV replication in human T cells. CypA knock-down in target human cells, which inhibited HIV-1 infection, did not reduce viral cDNA synthesis after SIV infection, indicating that target human cell CypA is essential for HIV-1 but not for SIV infection. These results imply different effects of CypA on HIV-1 and SIV replication in human T cells.

Our results revealed that CypA can be incorporated into SIV virions although not very efficiently. This is consistent with previous results [27,34]. A previous study implied inhibition of viral replication by over-incorporation of CypA in vif-deleted SIV [27], whereas the present study suggests that low levels of CypA proteins incorporated into wild-type SIV do not inhibit but may rather contribute to SIV replication in human T cells. Two amino acids in HIV-1 Gag, Gly221 and Pro222, were found to be important for the binding of CypA [21]. Although two amino acids are in fact conserved in our SIVagm isolate, the same region is not conserved in SIVmac239, which also was found to encapsidate CypA (Figure 1B). Therefore, it seems that CypA is incorporated into SIV virions through a mechanism that is distinct from HIV-1.

Target human cell CypA was shown to be essential for HIV-1 but not for SIV infection. This may partially explain different sensitivities of HIV-1 and SIV replication to CsA treatment, although other unknown host factors could also be involved in the CsA-mediated enhancement of SIV replication in human T cells. Indeed, CsA treatment enhanced SIV replication even in CypA-KD cells (Figure 4B). We also attempted to examine the effect of cyclophilin B (CypB), another CsA-sensitive PPIase, on SIV replication by knock-down of CypB (Figure 5). CypB knock-down showed no significant effect on HIV-1 replication but enhanced SIV replication in human T cells, suggesting a possible involvement of CypB in SIV replication. Overall SIV replication was enhanced in CypB-KD cells (Figure 5B, SIVagm) and CsA treatment resulted in only slight enhancement of SIV replication (data not shown). As predicted, HIV-1 replication was not affected by the CypB knock-down (Figure 5B, HIV-1) but was inhibited by CsA treatment (data not shown). Thus, enhanced replication of SIV in CsA-treated human cells as shown in Figure 1A might be best explained by the neutralization of CypB's inhibitory effect. Previous reports showed that CypB can bind to both HIV-1 and SIV CA [14,35], but CypB incorporation into HIV-1/SIV virions was undetectable in the present study (data not shown). Regarding the cellular localization of CypB, association of CypB with the endoplasmic reticulum (ER) via an ER signal sequence has been reported [36]. In other reports [37-39], CypB has been shown to be associated with heparan sulfate proteoglycans (HSPG) on the cell surface. Overall, it remains unclear how CypB affects SIV infection directly or indirectly, because most CypB is believed to express in the endoplasmic reticulum but not in cytoplasm [40].

In contrast, CsA treatment inhibited SIV (SIVmac239 and SIVagm) replication in macaque T cells; CsA treatment of either virus producer or target cells resulted in suppression of SIV replication. This CsA effect on SIV replication in macaque T cells is similar to that on HIV-1 replication in human T cells. Although our attempts to knock down CypA in macaque HSC-F cells were unsuccessful, we have obtained CypA knocked-down rhesus macaque kidney cell line: LLC-MK2 cells (Additional File 1). Analysis using these cells revealed that CypA is inhibitory for SIV replication in macaque cells and indicated that CypA dysfunction is likely to be largely involved in CsA-mediated reduction of SIV replication in macaque cells (Additional File 1). Taken together, the current study reveals that CsA treatment inhibited SIV replication in macaque T cells but enhanced SIV replication in human T cells, indicating a host species-specific effect of CsA on SIV replication. These data suggest that the effect of CsA on SIV infection seems to be general properties of SIV in human and macaque T cells, and cyclophilins may contribute to host-range control of certain lentiviruses.

Sequence analyses of CypA cDNA from human CEM-SS cells and macaque HSC-F, HSR-5.4, and Mn-3942 cells showed no difference in deduced amino acid sequences between human and macaque CypA (data not shown). Therefore, there may be a possible differential posttranslational modification of CypA such as acetylation [41,42] between human and macaque cells or there may be an additional host cell factor involved in the contribution of CypA to the determination of HIV-1 and SIV tropism by possibly affecting expression, localization, or function of CypA or viral capsid proteins.

CypA overexpression increased SIV infection in macaque T cells, suggesting that CypA may have different effects on SIV replication in human and macaque T cells. TRIM5α is known to restrict HIV-1 infection in macaque T cells, but this restriction has been shown to be relieved by CsA-mediated or small interfering RNA-mediated inhibition of CypA function, indicating involvement of CypA in TRIM5α-mediated restriction of HIV-1 infection in macaque cells [28,31,32]. Thus, CypA promotes HIV-1 infection in human cells but shows an inhibitory effect on HIV-1 infection in macaque cells. We attempted to examine the identification of macaque TRIM5α by PCR from macaque genomic DNA as reported previously [43] to investigate the possible effect of macaque TRIM5α on CsA-mediated reduction of SIV infection in macaque T cells, but macaque TRIM5α was identified in HSC-F cells but not in HSR-5.4 cells, suggesting that the effect of CsA on SIV replication in macaque T cells obtained from the present study (Figure 6) may be independent of macaque TRIM5α restriction (Additional File 2). In owl monkeys, which belong to new world monkeys, previous reports revealed the existence of a TRIM-CypA fusion protein (TRIMCyp) restricting HIV-1 infection, which was relieved by CsA treatment [44,45]. Recently, TRIMCyp has been found also in OWM, although OWM TRIMCyp did not restrict HIV-1 or SIVmac replication [43,46,47]. We also attempted to examine the identification of OWM TRIMCyp by PCR using primers on either side of the CypA insertion as previously reported [43] and the possibility of TRIMCyp expression by immunoblotting with anti-CypA antibody, but TRIMCyp expression was not detected in any of the macaque T-cell lines used in the present study (data not shown) although the CypA insertion was identified in the genome sequence of macaque T cells (Additional File 2).

Taken together, the present study reveals a species-specific effect of CsA on SIV replication. Our results suggest a contribution of CypA to efficient SIV replication in macaque cells. In contrast, analysis in human cells indicated that target cell CypA have no positive effect on SIV infection; rather, CypB was considered inhibitory for SIV replication. These results suggest possible involvement of cyclophilins in the determination of SIV tropism.

## Conclusions

The present study revealed that CsA treatment enhances SIV replication in human T cells but abrogates SIV replication in macaque T cells, indicating a host cell species-specific effect of CsA on SIV replication. CypA knock-down or overexpression indicated a positive effect of CypA on SIV infection into macaque but not into human T cells. These results suggest possible contribution of CypA to the determination of SIV tropism.

## Methods

### Analysis of SIV and HIV-1 replication in human and macaque T cells

HeLa cells were propagated in Dulbecco modified Eagles medium containing 10% fetal bovine serum (FBS). The human CEM-SS and A3.01 T cell lines were cultured in RPMI 1640 containing 10% FBS. Macaque peripheral blood mononuclear cells (PBMCs) and three macaque T-cell lines, cynomolgus macaque-derived HSC-F, rhesus macaque-derived HSR-5.4, and pig-tailed macaque-derived Mn-3942 were cultured in RPMI 1640 containing 10% FBS, 10 mM HEPES buffer, 50 μM 2-mercaptoethanol, and 10 U IL-2 per ml [48]. Virus stocks of SIVagm, SIVmac, and HIV-1 used for analysis of viral replication were prepared by transfection of HeLa cells using LipofectAMINE LTX PLUS (Invitrogen Corp., Carlsbad, CA) with molecular clone DNAs of SIVagm9063 [49], SIVmac239 [50], and HIV-1_NL4-3 _[51], respectively. An *env*-defective variant of SIVagm9063 carrying an insertion of stop codon in the *env *gene (nucleotide position 10-15 from start of the *env *gene) was constructed by site-directed mutagenesis. Titers of the virus stocks were quantitated by SIV CA (p27) or HIV-1 CA (p24) enzyme-linked immunosorbent assay (ZeptMetrix Corporation, Buffalo, NY) and by determining the reverse transcriptase (RT) activity. CEM-SS cells (5 × 10^5^) were exposed to 50 ng of SIV (p27) or 1 ng of HIV-1 (p24). Macaque T cells (5 × 10^5^) were incubated with 5 ng of SIV (p27), and rhesus PBMCs (5 × 10^5^) were incubated with 2 ng of SIV (p27). Virus production was monitored for 14 d post-infection by measuring RT activity in the culture supernatants as described previously [52]. In CsA treatment experiments, cells were cultured in the presence of 2.5 μM CsA (Sigma-Aldrich, Tokyo, Japan). Mean values in four independent experiments are shown.

### Immunoblot analysis

Immunoblot analysis of cell lysates and viral pellets was performed as described previously [53]. Briefly, virus supernatants containing equal RT levels were concentrated by centrifugation through 20% sucrose. Pelleted viruses were analyzed by immunoblotting using anti-CypA or anti-CA antibodies. A polyclonal anti-SIVagm CA antibody provided by Vanessa Hirsch [54], plasma from a SIVmac239-infected rhesus macaque, and a monoclonal anti-HIV-1 p24 antibody (Abcam Inc, Cambridge, MA) were used to detect SIVagm, SIVmac, and HIV-1 CA, respectively. We used polyclonal anti-CypA antibody (BIOMOL Research Laboratories Inc., Plymouth Meeting, PA) and monoclonal anti-α-tubulin antibody (Sigma-Aldrich). A representative result from four independent experiments is shown in each figure.

### Infectivity analysis

LuSIV cells which are derived from CEMx174 cells and contain a luciferase indicator gene under the control of the SIVmac239 LTR were maintained in RPMI 1640 medium containing 10% FBS and hygromycin B (300 μg/ml) [55]. LuSIV cells were cultured for 24 h after viral infection and lysed in 1 × reporter lysis buffer (Promega Corp., Madison, WI). To determine the luciferase activity, lysates were mixed with luciferase substrate (Promega Corp.) and light emission was measured in a luminometer (GloMax™ 96 Microplate Luminometer; Promega Corp.). Two sets of viruses were produced, and each was subjected to two sets of infection experiments. Thus, mean values of viral cDNA levels from four independent experiments are shown.

### Measurement of viral cDNA levels after viral entry

Viruses were prepared from virus-infected CEM-SS or HSC-F cells. For infection, 5 × 10^5 ^target cells were incubated with a SIVagm containing 500 ng of p27, SIVmac containing 500 ng of p27, or HIV-1 containing 10 ng of p24 for 24 hr, and then total cellular DNA was extracted using a DNeasy Tissue Kit (QIAGEN Inc., Valencia, CA). For CsA treatment of target cells, target cells were preincubated for 24 hr with 2.5 μM CsA (final concentration), a virus sample was added, and the infected cells (in 2.5 μM CsA, final concentration) were incubated for 24 hr. SIVagm, SIVmac, and HIV-1 inactivated by incubation at 65°C for 30 min were used as negative controls. For quantification of full-length viral cDNA levels by real-time PCR, primers 5'-GCTTCGGCCTCCATGATA-3' (nucleotides [nt] 1069-1086) and 5'-TGTTGCTACCGCTTCCTCTG-3' (nt 1231-1250) and probes 5'-TAGAACCAACAGGCTCGGAGGGCTTAAA-3' (nt 1141-1168) and 5'-AGTCTGTTCAATCTTGTGTGCGTGCTATATTGC-3' (nt 1170-1202) were used for amplification and detection of *gag *region of the SIVagm9063 genome (GenBank accession number L40990); primers 5'-GATCTCTCGACGCAGGACT-3' (nt 680-698) and 5'-CCCTGGCCTTAACCGAAT-3' (nt 844-861) and probes 5'-AGGCTAGAAGGAGAGAGATGGGTGCGAG-3' (nt 773-800) and 5'-GCGTCGGTATTAAGCGGGGGAGAATTAG-3' (nt 802-829) for amplification and detection of *gag *region of the HIV-1_NL4-3 _genome (GenBank accession number M19921); primers 5'-GTAGTATGGGCAGCAAATGA-3' (nt 1408-1427) and 5'-TGTTCCTGTTTCCACCACTA-3' (nt 1631-1650) and probes 5'-GCATTCACGCAGAAGAGAAAGTGAAACA-3' (nt 1568-1595) and 5'-ACTGAGGAAGCAAAACAGATAGTGCAGAGA-3' (nt 1597-1626) for amplification and detection of *gag *region of the SIVmac239 genome (GenBank accession number M33262). For quantification of full-length viral cDNA levels by real-time PCR in CypA-KD human T cells, primers 5'-AGTGGGAGTTTGTCAATACC-3' (nt 3787-3806) and 5'-CTGATTTGTTGTGTCCGTTAG-3' (nt 3954-3974) and probes 5'-AGATGGGGCAGCCAATAGGGAAACTAAATT-3' (nt 3875-3904) and 5'- GGAAAAGCAGGATATGTAACTGACAGAGGAAGACAA-3' (nt 3906-3941) were used for amplification and detection of *pol *region of the HIV-1_NL4-3 _genome. As the control for standardization, TaqMan Endogenous Control kit for glyceraldehyde-3-phosphate dehydrogenase (GAPDH) was used (Applied Biosystems, Inc., Foster City, CA). Real-time PCR was carried out in a LightCycler 2.0 instrument (Roche Diagnostics Corp., Indianapolis, IN). The ratios of viral cDNA levels to GAPDH DNA levels are shown.

### Establishment of CypA and CypB knocked-down human T cell lines

Both human CEM-SS and A3.01 T cells were transduced with HIV-1-based vectors that confer puromycin resistance and express short hairpin RNAs (shRNA) targeting human CypA and CypB (Sigma-Aldrich). CypA and CypB knocked-down cell lines were obtained after selection with 1 μg/ml puromycin. Representative results obtained from experiments using three CypA and CypB knocked-down human CEM-SS cell lines are shown.

### Exogenous expression of CypA in human and macaque T cells

CypA cDNAs amplified by PCR were inserted into the pMACS K^k^.HA(C) vector (Miltenyi Biotec GmbH, Bergisch Gladbach, Germany) to obtain pMACS K^k^.HA(C)-CypA expressing HA-tagged CypA (CypA-HA) together with a cytoplasmic domain-truncated mouse major histocompatibility complex class I H-2K^k ^protein, respectively. Human CEM-SS or macaque T cells (4 × 10^6 ^cells) suspended in 100 μl of nucleofection V solution with 2 μg of pMACS K^k^.HA(c)-CypA (CypA-HA) or pMACS K^k^.HA(c) (HA-control) plasmid vector DNAs were subjected to transfection (nucleofection) using a Nucleofector device. The nucleofection parameter was D-023 for CEM-SS cells and U-029 for HSC-F cells. After 1 day of culture, transfected cells were labeled with anti-H-2K^k ^microbeads (MACSelect K^k ^MicroBeads) and enriched by separation using the MACS Separator (Miltenyi Biotec GmbH). The enrichment rate of transfected cells was determined by detection of H-2K^k ^using MACSelect Control FITC Antibody (Miltenyi Biotec GmbH).

## Competing interests

The authors declare that they have no competing interests.

## Authors' contributions

HT conceived and coordinated the study, performed all analyses, and wrote the manuscript. HI assisted FACS analysis. NI and TK assisted infectious experiments. HA provided experimental tools. TM conceived and coordinated the study and was involved in writing the manuscript. All authors have read and approved the final manuscript.

## Supplementary Material

Additional file 1 **Effect of CypA knock-down on SIV infection in LLC-MK2 cells**. (A) Immunoblot analysis of CypA expression. Lysates of LLC-MK2 (normal) and CypA-KD cells were subjected to the immunoblot analysis using anti-α-tubulin, anti-CypA and anti-CypB antibodies (Abcam Inc., Cambridge, MA) (left panel). The image of one representative blot is shown. (B) Effect of CypA knock-down on SIV infection in LLC-MK2 cells. Normal and CypA-KD LLC-MK2 cells were transfected with plasmid SIVmac239LTR-luc that contains a luciferase indicator gene under the control of the SIVmac239 LTR. After 24 h, transfected cells were used for VSVG-pseudotyped SIVagm env(-) virus infection. Infection was determined 24 h later by measuring the Tat-induced luciferase activity in the transfected cells. Luciferase activity induced by the virus in normal LLC-MK2 cells was defined as 100%. Mean values and standard deviations in three independent experiments are shown. Click here for file

Additional file 2 **Identification of a TRIM5α or a TRIMCyp in macaque T cells**. Total DNA from macaque HSC-F and HSR-5.4 T cells was harvested. PCR primers on either side of the CypA insertion were used to detect both a TRIM5α and a TRIMCyp in macaque T cells as described [43]. H_2_O denotes water control. Click here for file
