# Supplementary figures and images for: Host cell species-specific effect of cyclosporine A on simian immunodeficiency virus replication
Source: Retrovirology. 2012 Jan 6;9:3. doi: 10.1186/1742-4690-9-3 (PMC3311600; doi:10.1186/1742-4690-9-3)

**A**

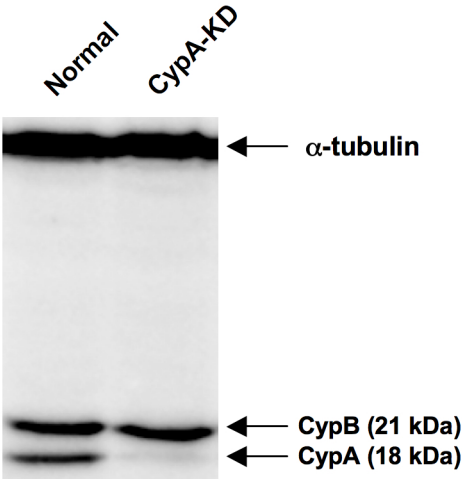

**B**

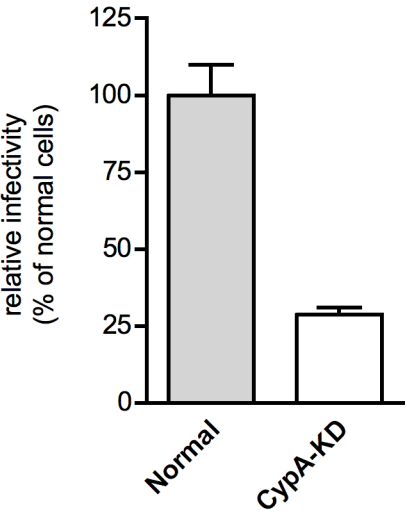

Supplement: Additional file 1 — Effect of CypA knock-down on SIV infection in LLC-MK2 cells. (A) Immunoblot analysis of CypA expression. Lysates of LLC-MK2 (normal) and CypA-KD cells were subjected to the immunoblot analysis using anti-α-tubulin, anti-CypA and anti-CypB antibodies (Abcam Inc., Cambridge, MA) (left panel). The image of one representative blot is shown. (B) Effect of CypA knock-down on SIV infection in LLC-MK2 cells. Normal and CypA-KD LLC-MK2 cells were transfected with plasmid SIVmac239LTR-luc that contains a luciferase indicator gene under the control of the SIVmac239 LTR. After 24 h, transfected cells were used for VSVG-pseudotyped SIVagm env(-) virus infection. Infection was determined 24 h later by measuring the Tat-induced luciferase activity in the transfected cells. Luciferase activity induced by the virus in normal LLC-MK2 cells was defined as 100%. Mean values and standard deviations in three independent experiments are shown. [file 1742-4690-9-3-S1.PDF]

Additional Figure 2

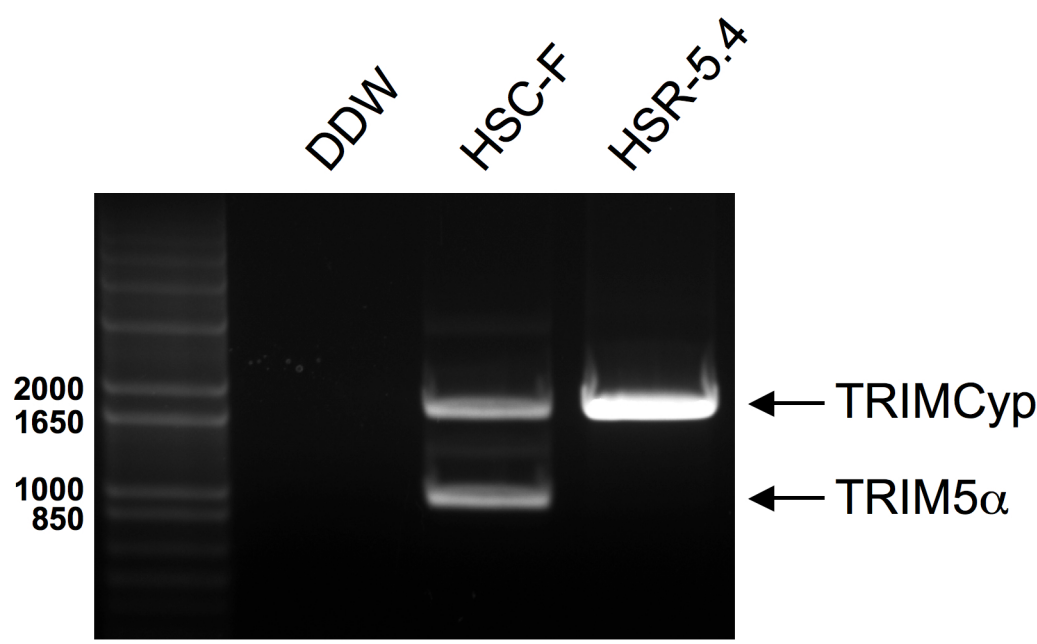

Supplement: Additional file 2 — Identification of a TRIM5α or a TRIMCyp in macaque T cells. Total DNA from macaque HSC-F and HSR-5.4 T cells was harvested. PCR primers on either side of the CypA insertion were used to detect both a TRIM5α and a TRIMCyp in macaque T cells as described [43]. H2O denotes water control. [file 1742-4690-9-3-S2.PDF]
